# RETRACTED: Atta et al. Effect of Montmorillonite Nanogel Composite Fillers on the Protection Performance of Epoxy Coatings on Steel Pipelines. *Molecules* 2017, *22*, 905

**DOI:** 10.3390/molecules31132290

**Published:** 2026-07-01

**Authors:** Ayman M. Atta, Ashraf M. El-Saeed, Hamad A. Al-Lohedan, Mohamed Wahby

**Affiliations:** 1 Chemistry Department, College of Science, King Saud University, Riyadh 11451, Saudi Arabia; hlohedan@ksu.edu.sa; 2 Petroleum Application Department, Egyptian Petroleum Research Institute, Nasr City, Cairo 11727, Egypt; ashrfelsaied@yahoo.com; 3 Arki for Advanced Construction Chemicals Co., Ltd., Riyadh 11451, Saudi Arabia; m.wahby@arkichem.com

The journal retracts the article titled “Effect of Montmorillonite Nanogel Composite Fillers on the Protection Performance of Epoxy Coatings on Steel Pipelines” [1], as cited above.

Following the publication, concerns were brought to the attention of the Editorial Office regarding the integrity and accuracy of the images and data presented in the published paper [1].

In accordance with the journal’s standard procedures, the Editorial Office and Editorial Board conducted an investigation that identified indications of inappropriate image editing and duplication between this article and a number of previously published works [2,3,4] by the similar authorship group. These concerns relate to the images and data presented in Figures 3, 7, and 9. Following further discussions with the authors, the Editorial Board were unable to verify the integrity of the figures in question based on the explanations and Supplementary Material provided. Consequently, the Editorial Board has lost confidence in the reliability of the overall findings and decided to retract this publication [1], as per MDPI’s retraction policy.

This retraction was approved by the Editor-in-Chief of the *Molecules* journal.

The authors have been informed of this retraction but did not provide a comment on this decision.

## References

[1] Atta A.M., El-Saeed A.M., Al-Lohedan H.A., Wahby M. (2017). RETRACTED: Effect of Montmorillonite Nanogel Composite Fillers on the Protection Performance of Epoxy Coatings on Steel Pipelines. Molecules.

[2] Wahby M.H., Atta A.M., Al-Lohedan H.A., El-saeed A.M., Tawfik A.M. (2017). Non-Cracked Epoxy Nanogel Composite as Anticorrosive Coatings for Aggressive Marine Environment. Int. J. Electrochem. Sci..

[3] Atta A.M., Al-Lohedan H.A., ALOthman Z.A., Tawfeek A.M., AbdelGhafar A., Hamad N.A. (2016). Effect of Zeta Potential of Exfoliated Amphiphilic Montmorillonite Nanogels on Removal Efficiencies of Cationic Dye Water Pollutant. Int. J. Electrochem. Sci..

[4] Atta A.M., El-Saeed A.M., Al-Shafey H.I., El-Mahdy G.A. (2016). Self-healing Passivation of Antimicrobial Iron Oxide Nanoparticles for Epoxy Nanocomposite Coatings on Carbon Steel. Int. J. Electrochem. Sci..

